# Results from a multicentre evaluation of plug use for left ventricular assist device explantation

**DOI:** 10.1093/icvts/ivab344

**Published:** 2021-12-09

**Authors:** Evgenij V Potapov, Nikolaos Politis, Matthias Karck, Michael Weyand, René Tandler, Thomas Walther, Fabian Emrich, Hermann Reichenspurrner, Alexander Bernhardt, Markus J Barten, Peter Svenarud, Jan Gummert, Davorin Sef, Torsten Doenst, Dmytro Tsyganenko, Antonio Loforte, Felix Schoenrath, Volkmar Falk

**Affiliations:** 1 Department of Cardiothoracic and Vascular Surgery, German Heart Center Berlin, Berlin, Germany; 2 German Center for Cardiovascular Research (DZHK), Partner Site Berlin, Berlin, Germany; 3 Abbott Medical GmbH, Eschborn, Germany; 4 Department of Cardiac Surgery, Heidelberg University Hospital, Heidelberg, Germany; 5 Department of Cardiac Surgery, University of Erlangen-Nuremberg, Erlangen, Germany; 6 Department of Cardiac Surgery, Johann-Wolfgang-Goethe University, Frankfurt, Germany; 7 Department of Cardiovascular Surgery, University Heart and Vascular Center Hamburg, Hamburg, Germany; 8 Department of Cardiothoracic Surgery, Karolinska University Hospital, Stockholm, Sweden; 9 Department of Thoracic and Cardiovascular Surgery, Heart and Diabetes Center NRW Ruhr-University of Bochum, Bad Oeynhausen, Germany; 10 Department of Cardiothoracic Transplantation and Mechanical Circulatory Support, Royal Brompton and Harefield NHS Foundation Trust, Harefield Hospital, London, UK; 11 Department of Cardiothoracic Surgery, Jena University Hospital, Friedrich Schiller University of Jena, Jena, Germany; 12 Division of Cardiac Surgery, IRCCS Azienda Ospedaliero-Universitaria di Bologna, S. Orsola University Hospital, Bologna, Italy; 13 Charité—Universitätsmedizin Berlin, corporate member of Freie Universität Berlin, Humboldt-Universität zu Berlin, and Berlin Institute of Health, Department of Cardiovascular Surgery, Berlin, Germany; 14 Department of Health Sciences and Technology, Eidgenössiche Technische Hochschule Zürich, Translational Cardiovascular Technology, Zurich, Switzerland

**Keywords:** MCS, Weaning, Recovery, Left ventricular assist device, Explantation, Plug

## Abstract

**OBJECTIVES:**

Myocardial recovery allows for left ventricular assist device (LVAD) explantations after long-term support. Several surgical approaches, including interventional decommissioning, off-pump explantation using a custom-made plug and complete LVAD removal through redo sternotomy, have been described. We present the results from an evaluation of the long-term follow-up of patients who received a titanium sintered plug after LVAD explantation.

**METHODS:**

We performed a retrospective, European, multicentre analysis of patients who received a titanium sintered plug to seal the apical fixation ring after LVAD explantation. Data were collected from a questionnaire that included demographics, procedural details and follow-up information.

**RESULTS:**

Out of 54 contacted centres in 12 countries (*n* = 179 patients), a total of 68 patients were successfully included in the study. The median follow-up was 34 months (interquartile range: 17–58.5 months); 57 (84%) patients had >1-year follow-up. At the time of the last follow-up, 55 (81%) patients were alive, with a Kaplan–Meier 1-year survival of 90.1% (95% confidence interval: 84.0–98.1%) and a 5-year survival of 80.0% (95% confidence interval: 68.4–92.9%). One patient (1.5%) developed a plug infection originating from an infected part of the incorporated driveline and, after complete removal, is currently in good condition. No postoperative stroke has been reported after plug implantation.

**CONCLUSIONS:**

In this European multicentre study, the use of a custom-made titanium plug to close the apical fixation ring after LVAD explantation resulted in a low incidence of plug-related complications. With the volume of patients undergoing LVAD explantations after myocardial recovery increasing, the plug has evolved as a simple alternative to more invasive device explantation procedures or decommissioning with a high risk for infection of the remaining system or stroke.

**Figure:**
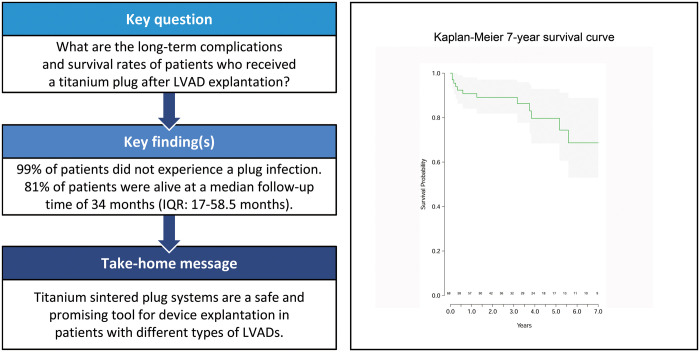

## INTRODUCTION

Left ventricular assist devices (LVADs) are implanted in patients with end-stage heart failure as a bridge to transplant, a bridge to recovery and a destination therapy. Several studies have demonstrated that myocardial recovery occurs after long-term LVAD support [1, 2]. The 2019 Interagency Registry for Mechanically Assisted Circulatory Support annual report states a recovery rate of 5% by 5 years [3]. Myocardial recovery may allow weaning and subsequent LVAD explantation [4]. Reported explantation rates from different centres vary from >50% in prospective trials on selected patients to 3.9% in a single centre [5] and 1.2% to 1.3% in large registries [6]. The number of patients undergoing LVAD implantation at an earlier stage of heart failure and the growing knowledge about mechanical support could increase the number of patients with the potential for myocardial recovery and subsequent LVAD explantation. Several reports have been published on the use of custom-made titanium plugs to facilitate the explantation of LVADs [7–9]. As the demand for such plug systems increases, a systematic outcome analysis is mandatory. We report the complication rate from an evaluation of the long-term follow-up of patients who received a titanium sintered plug to facilitate LVAD explantation.

## MATERIALS AND METHODS

### Ethics statement

Ethics committee approval (EA2/053/19) was granted from the Charité ethics committee prior to the beginning of the study. The study was reviewed and approved by an institutional review board or local ethics committee at each participating centre. No patient formal consent was obtained due to the anonymity of the data and the retrospective nature of the study.

**Figure 1:**
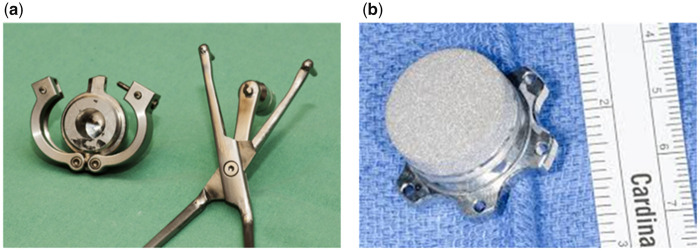
The recovery plug for a HeartMate 3 with a clamp for simplified plug insertion [9] (**a**) and a HeartWare HVAD plug [10] (**b**) (Fittkau Metallbau GmbH, Berlin, Germany).

The current evaluation was a retrospective, European, multicentre analysis. A request for data collection was sent out to hospitals that have used the titanium plug device. A total of 54 centres from 12 countries were contacted regarding the implantation of 179 titanium plug systems (Fittkau Metallbau GmbH, Berlin, Germany) between 2010 and 2021. The recovery plug system produced by Fittkau is a titanium sintered individually made plug that has 3 modifications for HeartMate II, HeartMate 3 and HeartWare HVAD, respectively (Fig. 1). The technical details of the plug system have been described elsewhere [7, 9, 10]. A comprehensive questionnaire was sent out to all centres, consisting of 6 multi-item domains labelled ‘Implantation information’, ‘Pre-LVAD explantation general parameters’, ‘Medication prior to explantation’, ‘Explantation information’, ‘Follow-up’ including the longest follow-up to date and the ‘Medication at follow-up’. The questionnaires were returned with the patient data collected pseudonymously using a uniquely identifiable study number. The study followed the Strengthening the Reporting of Observational studies in Epidemiology recommendations [11]. The goal of the study was to report the complication rate from the evaluation of the long-term follow-up in patients who received a titanium sintered plug to facilitate LVAD explantation.

### Statistical analyses

The data were entered in a Research Electronic Data Capture online database system for statistical analyses. A statistician performed all analyses using R software, version 4.02 (R Foundation for Statistical Computing, Institute for Statistics and Mathematics, Vienna, Austria). The data are presented as median [interquartile range (IQR)] for continuous variables and as frequency (percentage) for categorical variables. A Kaplan–Meier analysis was used to compute survival. The New York Heart Association (NYHA) functional classifications over time were graphically displayed in an alluvial diagram. NYHA values obtained at explant and at last follow-up were compared using the McNemar test. The impact of the NYHA value before explantation on survival was examined by Cox-regression analysis. The hazard ratio (HR) and a 95% confidence interval (CI) are provided.

## RESULTS

### Baseline characteristics

Data from 68 patients from 10 European centres, including the UK, that used titanium plugs for the explantation of an LVAD system, were successfully included in the study (Table 1). Following data were missing from the included patients; Interagency Registry for Mechanically Assisted Circulatory Support at baseline (*n* = 6), NYHA class before implantation (*n* = 1), device explantation procedure (*n* = 3) and NYHA at last follow-up (*n* = 2).

**Table 1:** Descriptive analysis of 68 patients at implantation

	Number of patients (*n* = 68)
Aetiology of CMP	
Idiopathic dilative	32 (47)
Acute myocarditis	11 (16)
Ischaemic	4 (6)
Toxic	9 (13)
Postpartum	3 (4.5)
Valvular	3 (4.5)
Other	6 (9)
INTERMACS at LVAD implantation^a^	
1	29 (47)
2	14 (22.5)
3	16 (26)
4	2 (3)
5–7	1 (1.5)
Type of LVAD	
HeartMate II	10 (15)
HeartMate 3	3 (4)
HeartWare HVAD	55 (81)
Median duration of long-term support in months	17.5 (IQR: 11.75-26.25)

*n* (%) if not otherwise specified.

a INTERMACS data were collected from only 62 patients.

CMP: cardiomyopathy; INTERMACS: Interagency Registry for Mechanically Assisted Circulatory Support; LVAD: left ventricular assist device.

### Explantation

The predominant reason for device explantation among the patients was elective explantation due to myocardial recovery in 49 (72%) patients, followed by urgent explantation due to driveline infection and/or thromboembolic events in 8 (12%) patients, respectively. The preferred access for LVAD explantation was a lateral thoracotomy in 54 (83%) patients and a median sternotomy in 11 (17%) patients [2, 7, 9]. Plug implantation was performed off pump in 45 (68%) cases; in 20 (29%) cases, with cardiopulmonary bypass and in 2 (3%) cases, extracorporeal life support was used. The usage and handling of the plug system were comparable for all LVAD device types (Videos 1 and 2).

No thromboembolic complications were reported after plug insertion. Postoperative anticoagulation varied among the centres. The most representative strategy for post-plug removal anticoagulation was a vitamin K-antagonist administered for 6 months with a target international normalized ratio of 2, followed by a low dose of acetylsalicylic acid to prevent clot formation on the plug surface if no other indication for continuation of anticoagulation existed (Table 2).

**Table 2:** Heart failure medication(s) at explantation and at the last follow-up

	Medication(s) at explantation	Medication(s) at last follow-up
Beta-blocker	58 (91)	25 (96)
ACE inhibitor	48 (75)	17 (65)
Angiotensin-converting enzyme inhibitor/ARB	13 (20)	5 (19)
Angiotensin neprilysin receptor blocker/ARNI	1 (2)	2 (8)
Aldosterone receptor blocker	53 (83)	14 (54)
Loop diuretic	31 (48)	14 (54)
Thiazide diuretic	3 (5)	0 (0)
Digoxin/digitoxin	4 (6)	2 (8)

*n* (%) if not otherwise specified.

ACE: angiotensin-converting enzyme: ARB: angiotensin receptor blockers; ARNI: angiotensin receptor neprilysin inhibitor.

### Follow-up

The date of the last follow-up was recorded for all patients included in the study. The median follow-up was 34 months (IQR: 17–58.5 months); 57 (84%) patients were followed for >1 year. Three patients (4%) were lost to follow-up.

**Figure 2:**
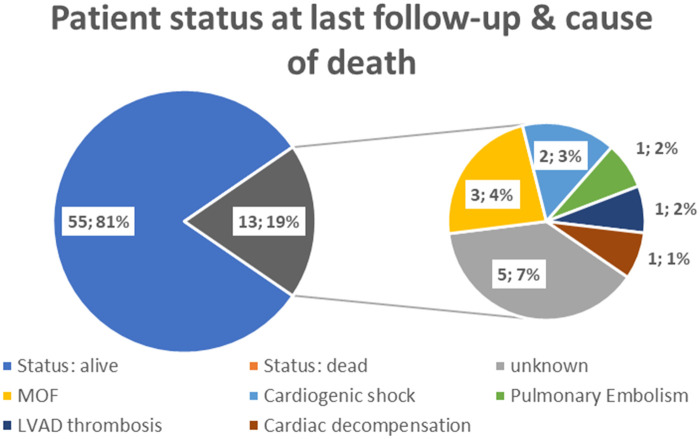
Patient status at the last follow-up and cause of death. LVAD: left ventricular assist device; MOF: multiple organ failure.

**Figure 3:**
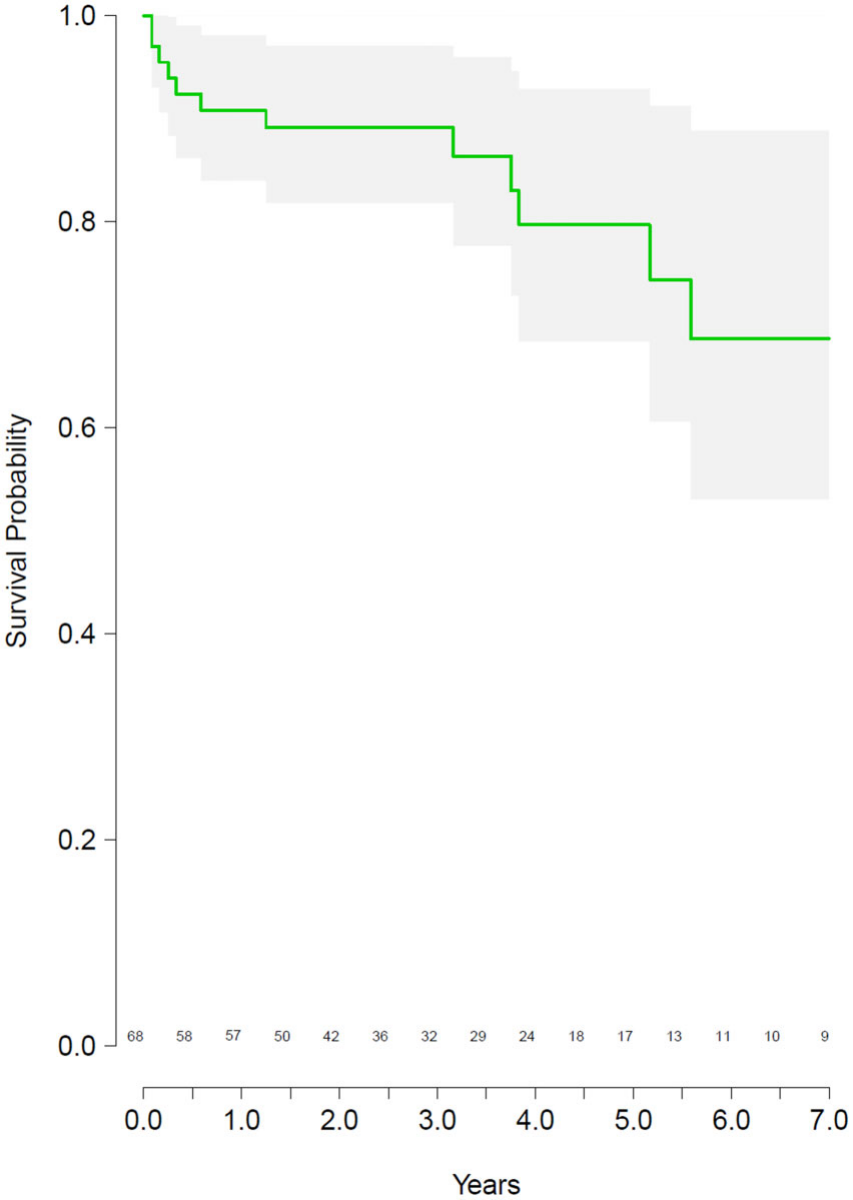
Kaplan–Meier survival curve.

**Figure 4:**
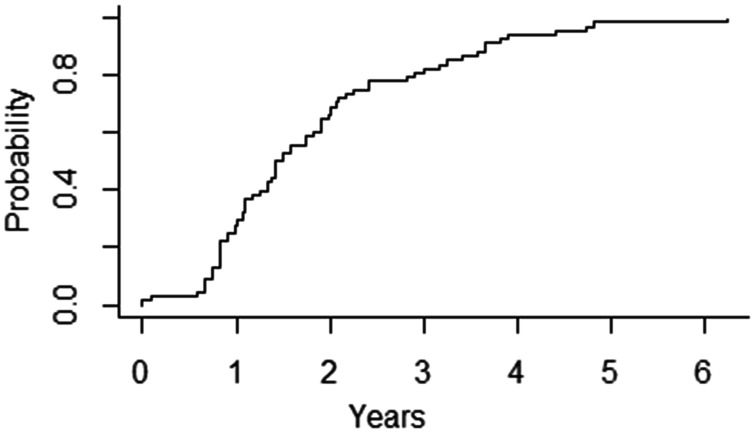
Cumulative incidence plot showing the time from implantation to explantation.

At the time of the last follow-up, 55 (81%) patients were alive. Thirteen (19%) patients died; the cause of death was unrelated to the plug system (Fig. 2). In 7 (54%) out of 13 patients who died, the ventricular assist device was explanted for urgent reasons (thromboembolic events, persistent gastrointestinal bleeding). The Kaplan–Meier analysis showed a 1-year survival of 90.1% (95% CI: 84.0–98.1%) and a 5-year survival of 80.0% (95% CI: 68.4–92.9%) (Fig. 3). Five patients (7%) underwent reimplantation of an LVAD system due to recurrent heart failure following explantation after a median of 13 months (IQR: 7–32.3 months), all with successful plug removal. During reimplantation and removal of the plug systems, no thrombotic material was detected. Overgrown tissue adhesions, due to the long implantation time of the plug, were cut to gain access to the left ventricular cavity. Two (3%) patients had a transplant following insertion of the plug. A cumulative incidence plot was drawn showing the time from implantation to explantation of the LVAD and insertion of the plug system (Fig. 4).

**Figure 5:**
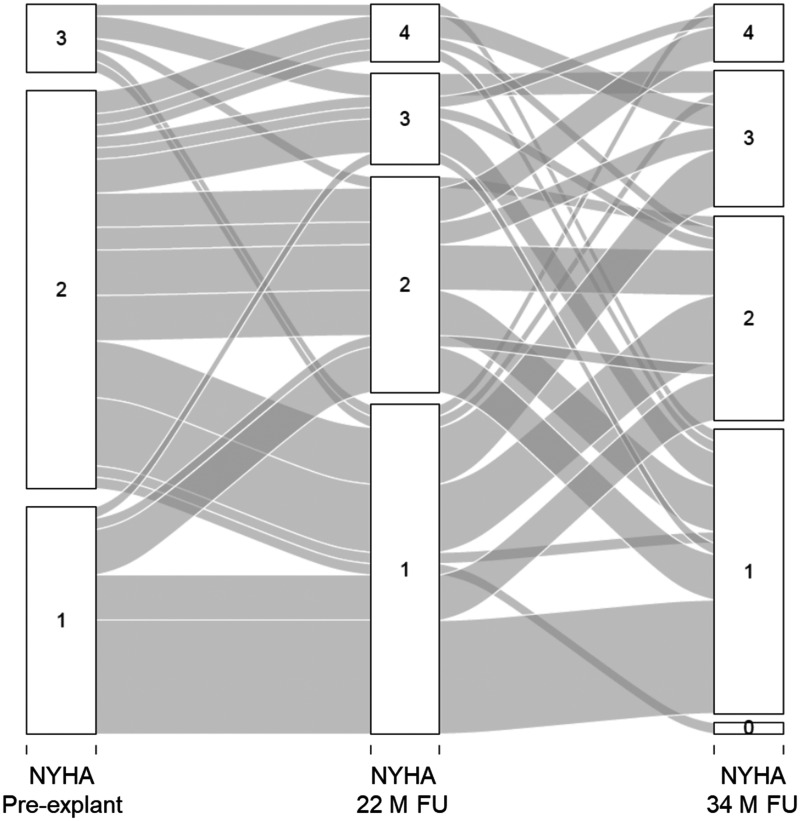
Progression of New York Heart Association functional class before explantation compared to New York Heart Association functional class at median follow-up of 22 months (interquartile range: 6-41 months) and median follow-up of 34 months (interquartile range: 17–58.5 months) per patient. NYHA: New York Heart Association; M: month; FU: follow-up; IQR: interquartile range.

Figure 5 graphically illustrates the NYHA functional classification over time in an alluvial diagram. The largest change in NYHA functional class happened from NYHA II pre-explantation compared to the last follow-up. Of 38 documented patients in NYHA functional class II pre-explantation, 11 patients (29%) improved to NYHA functional class I, 12 patients (32%) stayed the same, 8 patients (21%) worsened to NYHA functional class III, 4 patients (10%) worsened to NYHA functional class IV and 3 patients (8%) were undocumented at the last follow-up. The NYHA functional class at explantation was significantly different from the NYHA functional class at 34 months after explantation (*P* = 0.006).

The impact on survival of the higher NYHA functional class before explantation was significant: NYHA III versus NYHA I and NYHA II: HR 6.36; 95% CI: 1.51–26.81, *P* = 0.012. After the NYHA impact on survival was adjusted for duration on support and reason for explantation, NYHA III versus NYHA I and NYHA II, the HR was 9.12 and the 95% CI was 1.90–43.79, *P* = 0.006.

Only 1 patient (1.5%) developed a plug infection after LVAD explantation. The LVAD was explanted in November 2018 due to myocardial recovery and a concomitant driveline infection. During the explantation procedure, part of the cable could not be removed due to severe adhesions. The postoperative course was complicated by multiple revisions and finally by removal of the cable and titanium plug system including the fixation ring (HeartWare HVAD) and a reconstruction of the apex with a pericardial patch. To date, the patient is in stable condition with no signs of myocardial decompensation or infection.

## DISCUSSION

To our knowledge, the presented European multicentre study represents the largest detailed world-wide experience and the longest follow-up to date of scheduled and emergency LVAD explantations using plug systems. The main findings are as follows:

1. A total of 67 out of 68 (99%) patients who received a titanium plug did not experience a plug infection.
2. No postoperative stroke has been reported after plug implantation.
3. A total of 55 (81%) patients were alive at a median follow-up time of 34 months (IQR: 17–58.5 months).
4. Patients who died during follow-up had a higher NYHA functional class before explantation, indicating the necessity for a thorough evaluation to predict successful recovery.

Our data suggest that LVAD plug systems provide an easy and fast solution for LVAD explantation in the case of myocardial recovery with a low incidence of plug-related complications. Not only do plug systems facilitate durable LVAD explantations but could facilitate LVAD reinsertion should a patient develop recurrent heart failure following device explantation for recovery.

An increasing number of anecdotal success cases of LVAD weaning and explantation have been reported lately [1, 12–15]. The incidence of myocardial recovery and subsequent LVAD explantation in young patients with dilative cardiomyopathy or acute myocarditis and with shorter durations of heart failure is increasing, with a rate of 5–10% [5, 7] and up to 50% in strictly selected patients with optimal heart failure medication [1]. Large prospective studies to facilitate myocardial recovery during LVAD support are currently underway. Therefore, an optimal explantation strategy with a good safety profile and a low risk for complications is needed. Various methods for LVAD explantations currently exist. One of the options is an invasive reoperation for complete device removal and patch repair of the apex [16]. The major issues remain the inherent risks of bleeding, cardiac injury, right heart failure and complications that follow such an invasive procedure [15, 16]. Decommissioning is mostly performed as an operative procedure with infection rates of up to 41%, leading to death or complete device explantation in most patients [17]. Avoiding operative procedures by an interventional occlusion of the outflow graft may reduce the infection rate. However, the cable (including the biofilm), which is the source of infection, remains in situ during decommissioning, whereas it is completely removed during pump explantation or use of a titanium plug. Interventional percutaneous decommissioning may be considered for non-infected LVAD devices. However, because the driveline and inflow cannula remain in place when the LVAD is abandoned, patients are at high risk of infection or thrombus formation on the inflow cannula and are required to take life-long anticoagulation agents following this procedure [17, 18].

Nevertheless, interventional procedures appear to be an alternative to invasive LVAD explantation in sick patients with a high risk for surgery [5, 12, 13]. The minimally invasive off-pump LVAD removal, using a custom-made plug, is an optimal solution with a good safety profile and favourable long-term outcomes. However, in the presence of pump infection involving the fixation ring, the plug system should not be used.

During the duration of the study, only plug systems from Fittkau Metallbau were available for use. However, recently, a case report using a similar plug for HeartMate 3 removal was published showing that the plug concept is gaining more acceptance [8].

### Limitations

The plug systems are not yet CE-marked and can therefore only be used off-label. In Europe, the current process for plug use approval requires that one submit a request to the country-specific authorities on a patient-by-patient basis. This process may deter some from using this device for LVAD removal. The study has a low response rate based on its retrospective and voluntary nature. Nevertheless, data from more than one-third of the plug systems used were collected and analysed. The current study is also limited by the absence of some baseline patient characteristics, such as age or gender. However, the study was focused on follow-up after plug insertion rather than on predictors of myocardial recovery from LVAD. In follow-up studies, data on antibiotic treatment regimens and comparisons of the anticoagulation protocols of the participating centres would be beneficial. Such data, as well as new plug systems developed by LVAD manufacturers, are therefore needed to provide a safe and easy device explantation option that is compliant with all regulatory requirements.

## CONCLUSION

The results from our retrospective European study suggest that custom-made titanium plugs are a safe and promising tool for device explantation in patients with different types of LVADs. The favourable long-term survival outcomes and low complication rates reported in this study further strengthen the plug’s position as a rising alternative to standard device explantation procedures.

**Conflict of interest:** Evgenij V. Potapov reports institutional fees for consultancy, lectures and research activity and proctoring support from Abbott Medical GmbH, institutional fees for consultancy, lectures and research activity, proctoring and advisory board member activities from Medtronic GmbH, outside the submitted work. Alexander Bernhardt reports personal fees from Abbott, personal fees from Abiomed, personal fees from AstraZeneca, personal fees from Berlin Heart, personal fees from Medtronic and personal fees from Novartis, outside the submitted work. Markus J. Barten reports personal fees from Medtronic and personal fees from Abbott, outside the submitted work. Felix Schoenrath reports institutional fees for consultancy, lectures and research activity support from Abbott GmbH, travel support from Medtronic GmbH, institutional research support from Cardiorentis AG, institutional fees for lectures from Orion Pharma GmbH and institutional fees for lecturesfrom Astra Zeneca, outside the submitted work. Volkmar Falk reports eductional grants from Medtronic, from Abbott, from Boston Scientific, from Edwards Lifesciences and from JOTEC/CryoLife; research & study funds support from Biotronik and Berlin Heart and from Novartis Pharma, outside the submitted work; In addition, Volkmar Falk has a patent and a patent application, /Implantable pump system, as well as a method for bringing a pump system to a location application’. All other authors reported no conflicts of interest. 

## Data Availability Statement

The data underlying this article will be shared on reasonable request to the corresponding author. The data are stored on an institutional database, physically in Berlin, Germany.

## Author contributions

**Evgenij V. Potapov:** Conceptualization; Investigation; Methodology; Project administration; Resources; Supervision; Validation; Writing—original draft; Writing—review & editing. **Nikolaos Politis:** Conceptualization; Data curation; Formal analysis; Methodology; Visualization; Writing—original draft; Writing—review & editing. **Matthias Karck:** Investigation; Writing—review & editing. **Michael Weyand:** Investigation; Writing—review & editing. **René Tandler:** Investigation; Writing—review & editing. **Thomas Walther:** Investigation; Writing—review & editing. **Fabian Emrich:** Investigation; Writing—review & editing. **Hermann Reichenspurrner:** Investigation; Writing—review & editing. **Alexander Bernhardt:** Investigation; Writing—review & editing. Markus J. Barten**:** Investigation; Writing—review & editing. Peter Svenarud**:** Investigation; Writing—review & editing. **Jan Gummert:** Investigation; Writing—review & editing. **Davorin Sef:** Investigation; Writing—review & editing. **Torsten Doenst:** Investigation; Writing—review & editing. **Dmytro Tsyganenko:** Investigation; Writing—review & editing. **Antonio Loforte:** Investigation; Writing—review & editing. **Felix Schoenrath:** Conceptualization; Formal analysis; Methodology; Writing—original draft; Writing—review & editing. **Volkmar Falk:** Conceptualization; Resources; Supervision; Writing—original draft; Writing—review & editing.

## Reviewer information

Interactive CardioVascular and Thoracic Surgery thanks Guillaume Coutance, Daniel Y. Loisance and the other, anonymous reviewer(s) for their contribution to the peer review process of this article.

## ABBREVIATIONS

CI: Confidence interval
HR: Hazard ratio
IQR: Interquartile range
LVAD: Left ventricular assist device
NYHA: New York Heart Association
